# Convenient synthesis of dipeptide structures in solution phase assisted by a thioaza functionalized magnetic nanocatalyst

**DOI:** 10.1038/s41598-022-07303-3

**Published:** 2022-03-18

**Authors:** Reza Taheri-Ledari, Fereshteh Rasouli Asl, Mahdi Saeidirad, Amir Kashtiaray, Ali Maleki

**Affiliations:** Catalysts and Organic Synthesis Research Laboratory, Department of Chemistry, Iran University of Science and Technology, Tehran, 16846-13114 Iran

**Keywords:** Catalyst synthesis, Catalytic mechanisms, Organocatalysis

## Abstract

In this study, a heterogeneous nanocatalyst is presented that is capable to efficiently catalyze the synthetic reactions of amide bond formation between the amino acids. This nanocatalyst which is named Fe_3_O_4_@SiO_2_/TABHA (*TABHA stands for thio-aza-bicyclo-hepten amine*), was composed of several layers that increased the surface area to be functionalized with 2-aminothiazole rings via Diels–Alder approach. Firstly, various analytic methods such as Fourier-transform infrared (FTIR) and energy-dispersive X-ray (EDX) spectroscopic methods, thermogravimetric analysis (TGA), electron microscopy (EM), and UV–vis diffuse reflectance spectroscopy (UV-DRS) have been used to characterize the desired structure of the Fe_3_O_4_@SiO_2_/TABHA catalyst. Afterward, the application of the presented catalytic system has been studied in the peptide bond formation reactions. Due to the existence of a magnetic core in the structure of the nanocatalyst, the nanoparticles (NPs) could be easily separated from the reaction medium by an external magnet. This special feature has been corroborated by the obtained results from vibrating-sample magnetometer (VSM) analysis that showed 24 emu g^−1^ magnetic saturation for the catalytic system. Amazingly, a small amount of Fe_3_O_4_@SiO_2_/TABHA particles (0.2 g) has resulted in ca. 90% efficiency in catalyzing the peptide bond formation at ambient temperature, over 4 h. Also, this nanocatalyst has demonstrated an acceptable recycling ability, where ca. 76% catalytic performance has been observed after four recycles. Due to high convenience in the preparation, application, and recyclization processes, and also because of lower cost than the traditional coupling reagents (like TBTU), the presented catalytic system is recommended for the industrial utilization.

## Introduction

In recent decades, small metal-free organic molecules with the catalytic activity (called as organocatalysts), have been highly noticed by the researchers in the field^1,2^. This type of organic compounds include an active chemical site in their structures, which are able to create effective interactions such as hydrogen bond and electrostatic interactions with the raw materials^3^. As the main disadvantage for the organocatalysts, homogeneity can be referred, which creates requirements for the complex work up procedures^4^. Hence, the catalytic approaches turned into the use of the heterogeneous catalytic systems^5–7^. As one of the most well-known species of the heterogeneous catalytic systems, functionalized magnetic nanoparticles (MNPs) (known as magnetic nanocatalysts) have been widely used in different reactions^8–10^. In this type of materials, the organic structures (including the active sites) are loaded onto the surface of the heterogeneous MNPs via covalent bonding^11,12^. After completion of the catalytic process, the nanocatalyst particles are conveniently separated from the reaction mixture through holding an external magnet at the bottom of the flask. As another excellence of the nanocatalysts, high surface to volume ratio that can intensify the interactions between the reactants and catalyst is mentioned. Utilization of the MNPs as a heterogeneous substrate for immobilization of organocatalysts can provide other brilliant advantages such as successive recyclization and reuse^13–15^, hybridization with other compounds^16–18^, and application of auxiliaries (like ultrasound waves) than the homogeneous analogues^19–21^. Furthermore, the ability to modify the surface of these nanocatalysts with different organic compounds or amorphous structures like silica network, as a secondary shell, is another advantage of this type of systems^22,23^. The external shells can isolate the magnetic cores and protect them in the high-temperature processes. Silica, which is commonly employed as a support material in core–shell structures, not only enhances the stability of the nanoparticles (NPs) in a certain condition, but also allows them to be readily modified with various functional groups^24,25^.

Concerning peptide’s key role in the living organisms, they are of a great importance in chemistry, biochemistry, and pharmaceutical researches^26–28^. In this regard, peptide-drug conjugates (PDCs) represent a new generation of the high-tech pharmacy with high efficacy^29–31^. This prodrug strategy uniquely and specifically takes advantage of the biological activities and self-assembling potential of short-chain peptides to enhance the therapeutic efficacy of the medicinal compounds. In the field of peptide–drug conjugation, we have to deal with amide bond formation between chemical compounds and biological structures that are mostly made of protein strands and amino acid units^32,33^. In the same context, antibody–drug conjugates as a new generation of anticancer drugs with high efficiencies are highly noticed by the researchers^34^. This is why, development of the novel peptide coupling reagents with the economic benefits (like reusable heterogeneous species) is of high importance^35,36^.

One of the mostly noticed subjects in the field of organic synthesis, is development of the novel approaches that enable the creation of cyclic compounds from readily available starting materials with high selectivity, operational simplicity, functional-group tolerance, and environmental friendliness^37,38^. Among the well-known cyclization reactions in organic chemistry, Diels–Alder (D–A) reaction is one of the most fundamental and synthetically useful methods, which has been considered in chemistry and biochemistry as a simple one-step mechanism^39–41^. In this mechanism, a six-membered ring is formed through a [4 + 2] cycloaddition reaction, which serves a versatile tool for direct attachment of the organic molecules onto the surface of the nanoscale particles^42,43^.

In this study, an attempt was made to design a new and convenient method for catalytic reactions of peptide bond formation by the use of a core/shell structure made of iron oxide MNPs, silica network, and a thio-aza-bicyclo-hepten amine (TABHA) compound, formulated as “*Fe*_*3*_*O*_*4*_*@SiO*_*2*_*/TABHA*”. In order to build the Fe_3_O_4_@SiO_2_/TABHA catalytic system, silica-coated MNPs were prepared via a co-deposition method, and further modified by a vinylsilane compound. Then, the vinyl-functionalized Fe_3_O_4_@SiO_2_ MNPs were considered as a substrate for supporting 2-aminothiazole through the Diels–Alder reaction. The fabricated Fe_3_O_4_@SiO_2_/TABHA catalytic system has shown high catalytic proficiency in the peptide bond formation reactions, where ca. 90% reaction yield was obtained in only 4 h. Moreover, it has been observed that only 14% of its catalytic performance is reduced after four successive recycles and reuse, corroborating the heterogeneity and high structural stability of the system. Fe_3_O_4_@SiO_2_/TABHA is easily separated from the reaction medium via holding an external magnet at the end of the reaction flask. Overall, utilization of the presented nanocatalyst in recommended for high scale synthesis due to its economic benefits and considerable efficiency.

## Results and discussion

### Preparation of Fe_3_O_4_@SiO_2_/TABHA nanoparticles

**Figure 1:**
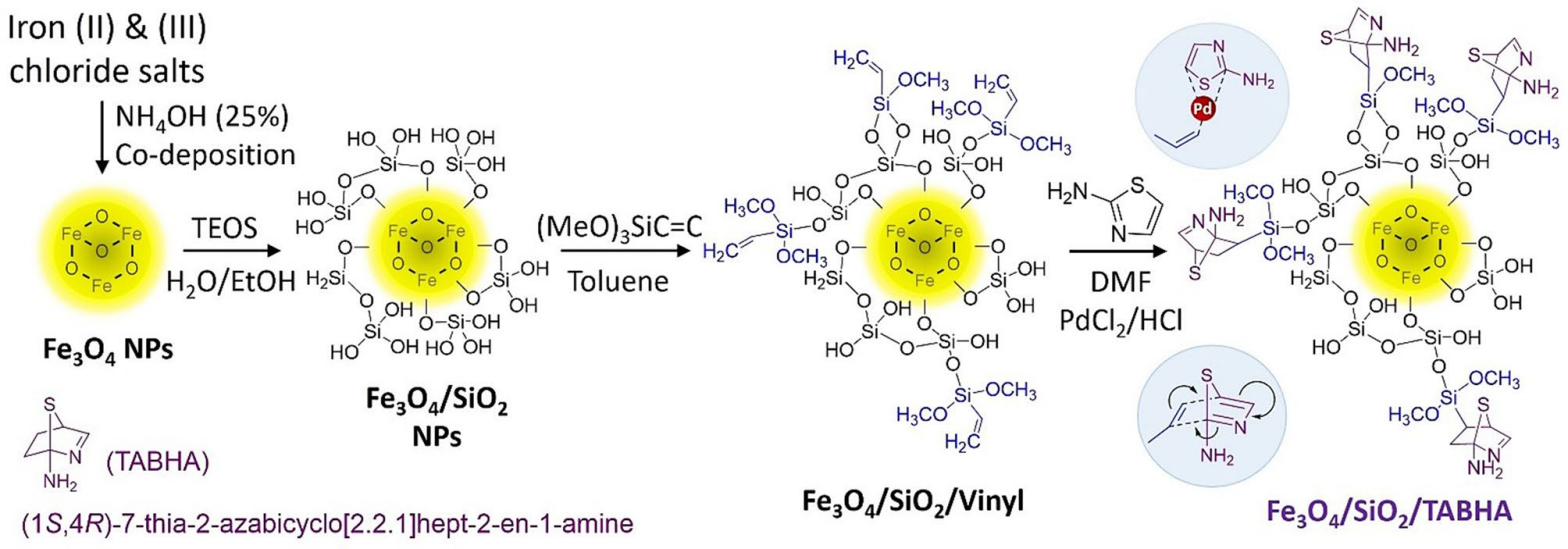
Schematic presentation of preparation route of Fe_3_O_4_@SiO_2_/TABHA catalytic system via Diels–Alder reaction approach.

As shown in Fig. 1, several steps are taken to prepare the Fe_3_O_4_@SiO_2_/TABHA nanoparticles (NPs), in which Fe_3_O_4_ is synthesized via co-deposition method using iron (II) and iron (III) chloride salts in a basic condition (pH ~ 12)^44–46^. Then, in order to place multiple hydroxyl groups on Fe_3_O_4_ MNPs, tetraethyl orthosilicate (TEOS) was used to coat the surface of the magnetic cores (SiO_2_ shell)^47,48^. In the next step, the surface of Fe_3_O_4_@SiO_2_ core/shell MNPs was functionalized with vinyl groups, by using trimethoxy vinylsilane (TMVS)^49^. In the final stage, the produced Fe_3_O_4_@SiO_2_@vinyl MNPs entered into a reaction with 2-aminothiazole in the presence of palladium (II) chloride, which leads to a Diels–Alder reaction on the surface of MNPs^50^.

In order to reach the optimized conditions for the synthesis of the Fe_3_O_4_@SiO_2_/TABHA catalytic system, different amounts of the particles in different solvents, at different temperatures and with different amounts of silver nitrate were carefully monitored. The details of this investigations are reported in Table 1. We considered the weight percentage of the sulfur atom in the EDX spectra as a criterion of the loading ratio in each product. The maximum ratio of the loading of 2-aminothiazole was obtained under reflux condition in dimethylformamide (DMF) at 110 °C, using 0.05 g of AgNO_3_. It has been distinguished by the superscript letter “c” in the Table 1.

**Table 1:** The obtained results from the optimization of the Diels–Alder Reaction onto the surface of Fe_3_O_4_@SiO_2_@vinyl MNPs.

Entry	Method	Solvent	AgNO_3_ (mg)	Time (h)	Sulfur (wt%)^a^
1	Reflux	W/P^b^	–	24	0.20
2	Reflux	W/P	25	24	0.43
3	Reflux	W/P	50	24	0.53
4	Reflux	DMF	25	12	1.01
5	Reflux	DMF	50	12	1.03^c^
6	Reflux	W/E^d^	50	12	0.52
7	Autoclave	DMF	50	12	0.49
8	Autoclave	DMF	50	24	1.02
9	Ultrasonication	W/P	50	1	0.28
10	Ultrasonication	W/P	50	3	0.37

^a^Wt% of S was obtained using EDX analysis.

^b^Water–PEG-200 [1:1].

^c^Optimum condition.

^d^Water–ethanol [1:1].

### Characterization of Fe_3_O_4_@SiO_2_/TABHA nanoparticle

#### FTIR spectroscopy

**Figure 2:**
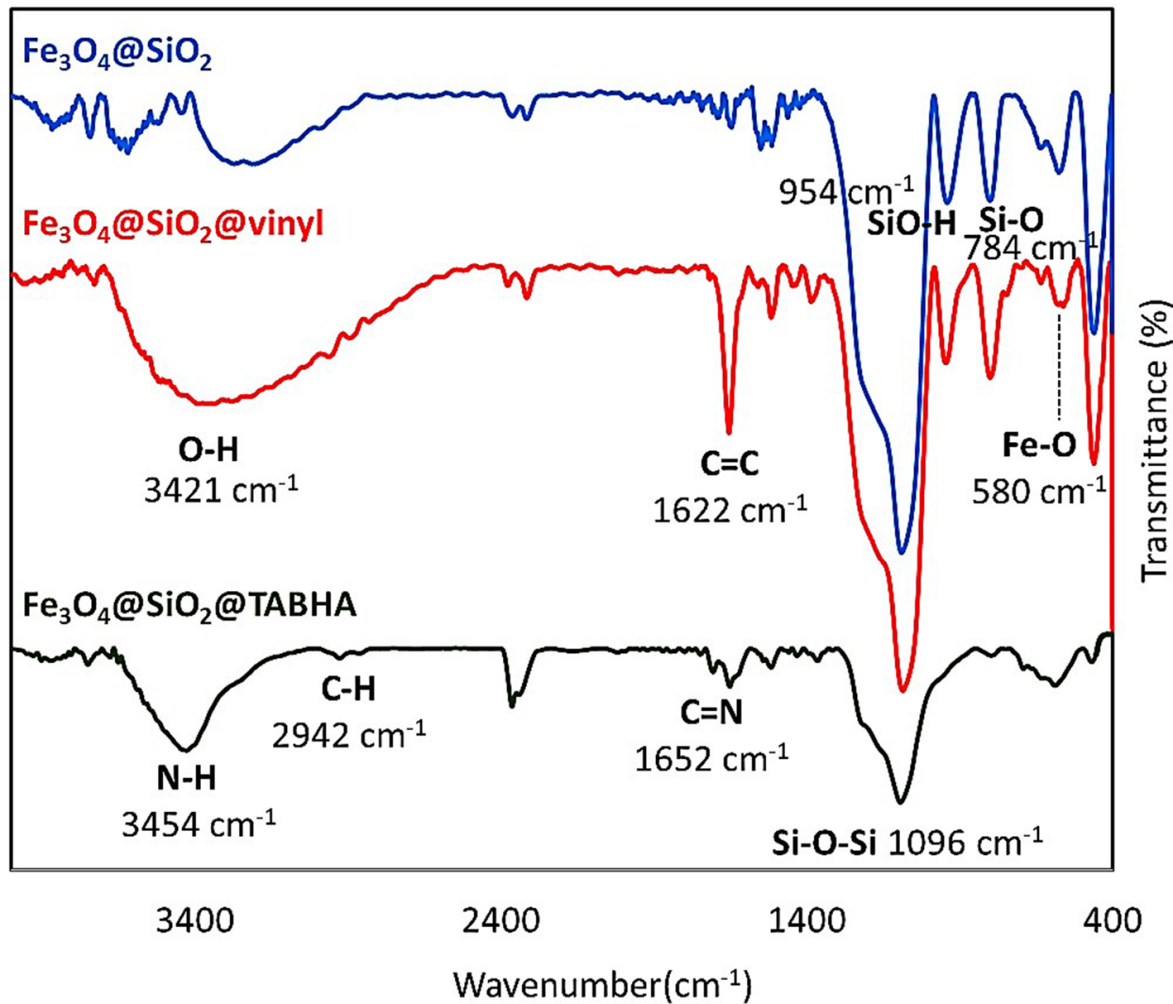
The FTIR spectra of Fe_3_O_4_@SiO_2_ (blue), Fe_3_O_4_@SiO_2_@vinyl (red), and Fe_3_O_4_@SiO_2_/TABHA nanoparticle (jaspery).

In order to examine the functional groups of the produced materials, Fourier-transform infrared (FTIR) spectrum was checked for Fe_3_O_4_@SiO_2_/TABHA nanoparticle, as shown in the Fig. 2. The presence of Fe–O, Si–O, Si–OH, and Si–O–Si bonds, has been confirmed in all samples by the appeared peaks at 580, 784, 954, and 1096 cm^−1^, respectively^51,52^. For Fe_3_O_4_@SiO_2_@vinyl sample, the peak appeared at 1622 cm^−1^ is related to C=C stretching^53^. Also, in the same case, the signal related to C–H bonds (sp2) is covered by the broad peak of the –OH groups that appeared at 3000–3100 cm^−1^^54^. In the spectrum of Fe_3_O_4_@SiO_2_/TABHA NPs, there are new peaks appeared at 1652, 3454, and 2942 cm^−1^ that are attributed to the stretching vibrations of C=N^55^, free NH_2_^56^, and C–H bonds (hybridation sp3)^57^, respectively. These new peaks prove the formation of Fe_3_O_4_@SiO_2_/TABHA NPs in terms of FTIR.

#### EDX analysis

**Figure 3:**
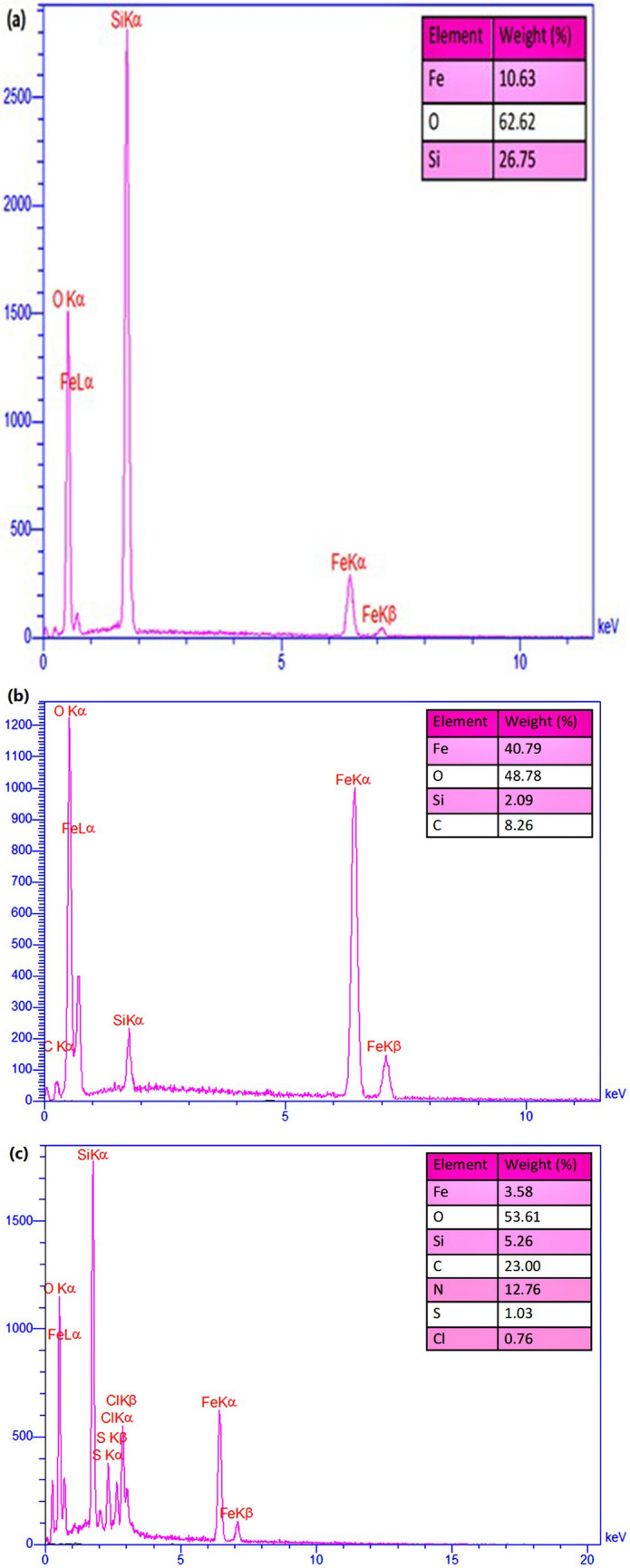
EDX spectra of (**a**) Fe_3_O_4_@SiO_2_, (**b**) Fe_3_O_4_@SiO_2_@vinyl, and (**c**) Fe_3_O_4_@SiO_2_/TABHA nanoparticle.

Energy-dispersive X-ray (EDX) spectroscopy was used to further confirm the existence of the elements that are predicted to be present after completion of each stage of preparation. As shown in Fig. 3, all of the composition elements for three materials including Fe_3_O_4_@SiO_2_, Fe_3_O_4_@SiO_2_@vinyl and Fe_3_O_4_@SiO_2_/TABHA samples, were detected and confirmed through the EDX peaks. The provided spectra show the presence of Fe, Si, O, and C elements after performing the coating reactions. The presence of carbon element in Fig. 3b is an evidence for successful loading of TMVS on the surface of Fe_3_O_4_@SiO_2_ NPs. Also, the Fig. 3c clearly demonstrates that 1.03 wt% of the total weight of the fabricated Fe_3_O_4_@SiO_2_/TABHA nanoparticle belongs to the sulfur atom, which can be a sign for the correct synthesis of the desired catalyst. The remarkable point about the EDX of Fe_3_O_4_@SiO_2_/TABHA nanoparticle is that this analysis shows the presence of Cl while none of the ingredients of this nanoparticle have Cl. The reason for the presence of Cl in the EDX analysis of Fe_3_O_4_@SiO_2_/TABHA nanoparticle is that one of the synthesis steps of this nanoparticle is performed by the Diels–Alder reaction, which is catalyzed by PdCl_2_, so a small number of Cl ions released by PdCl_2_ still remains in the porous structure of SiO_2_ of this nanoparticle and has not been removed even after several rinsing the Fe_3_O_4_@SiO_2_/TABHA NPs.

#### Electron microscopy

**Figure 4:**
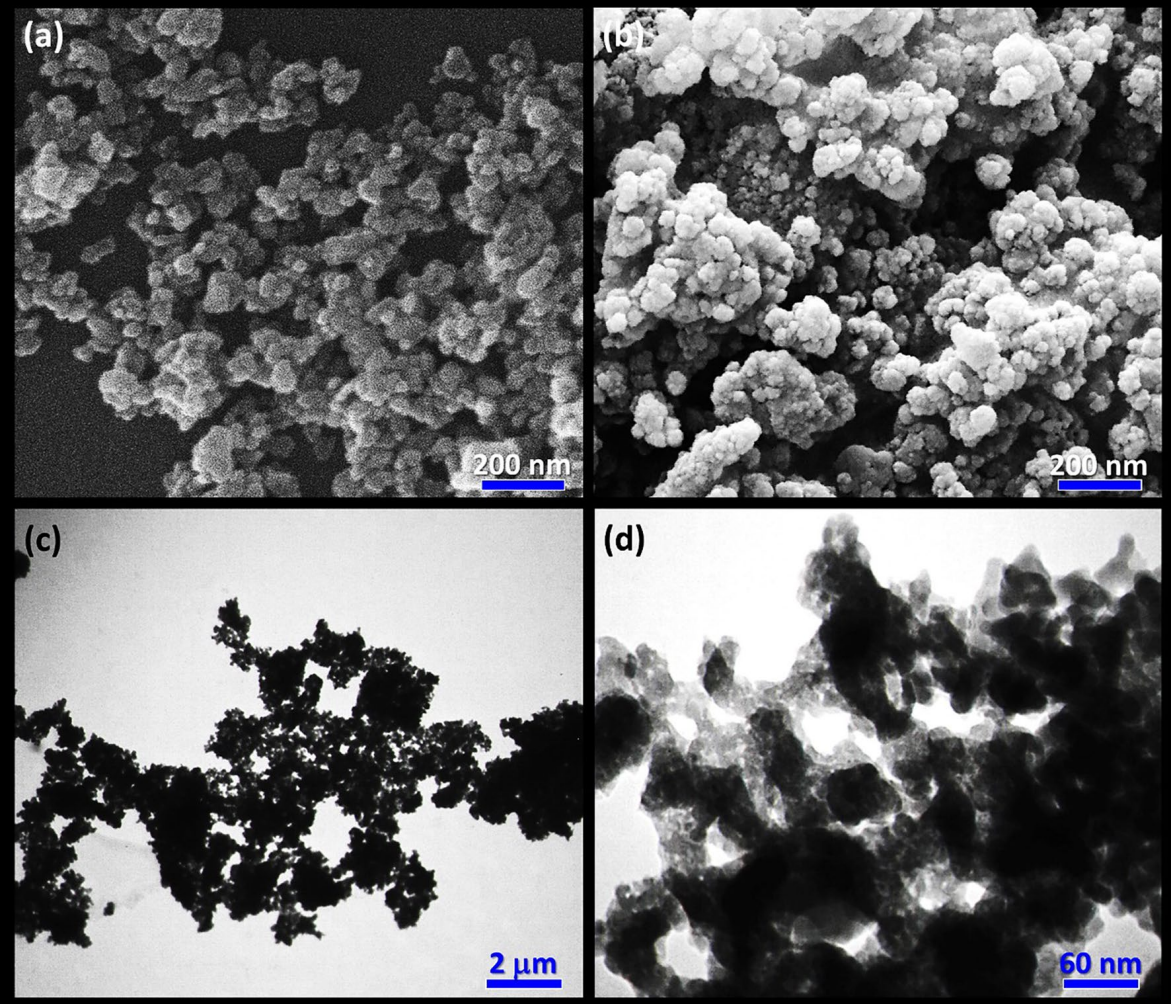
SEM images of (**a**) Fe_3_O_4_ NPs, (**b**) Fe_3_O_4_@SiO_2_/TABHA NPs, and (**c**,**d**) TEM images of Fe_3_O_4_@SiO_2_/TABHA NPs.

In order to investigate morphologies, real structures, sizes, and other properties of the prepared Fe_3_O_4_@SiO_2_/TABHA nanoparticles, scanning-electron microscopy (SEM) and transmission-electron microscopy (TEM) were used. As illustrated in Fig. 4a,b, the Fe_3_O_4_@SiO_2_/TABHA MNPs exhibited highly dispersed particles with a spherical morphology. Good dispersion of the Fe_3_O_4_@SiO_2_/TABHA NPs provides an extremely active surface area for the catalytic applications. The dispersion state of the Fe_3_O_4_@SiO_2_/TABHA NPs was also investigated by dynamic-light scattering (DLS) analysis. As shown in Fig. S1 (in the SI section), the mean size of the particles was estimated to be 74.5 nm, with a poly-dispersity index of 1.2. The size of the NPs in Fig. 4b, which are related to the Fe_3_O_4_@SiO_2_/TABHA sample, is larger than the neat Fe_3_O_4_ NPs, shown in Fig. 4a. This difference in size indicates that additional layers have been formed around the Fe_3_O_4_ magnetic core. Furthermore, the provided TEM images from Fe_3_O_4_@SiO_2_/TABHA NPs (Fig. 4c,d) reveal that the core/shell structure has been properly constructed. In these images, the black areas are related to the magnetic cores (Fe_3_O_4_), and the gray areas are related to the shell (SiO_2_/TABHA).

#### VSM analysis

**Figure 5:**
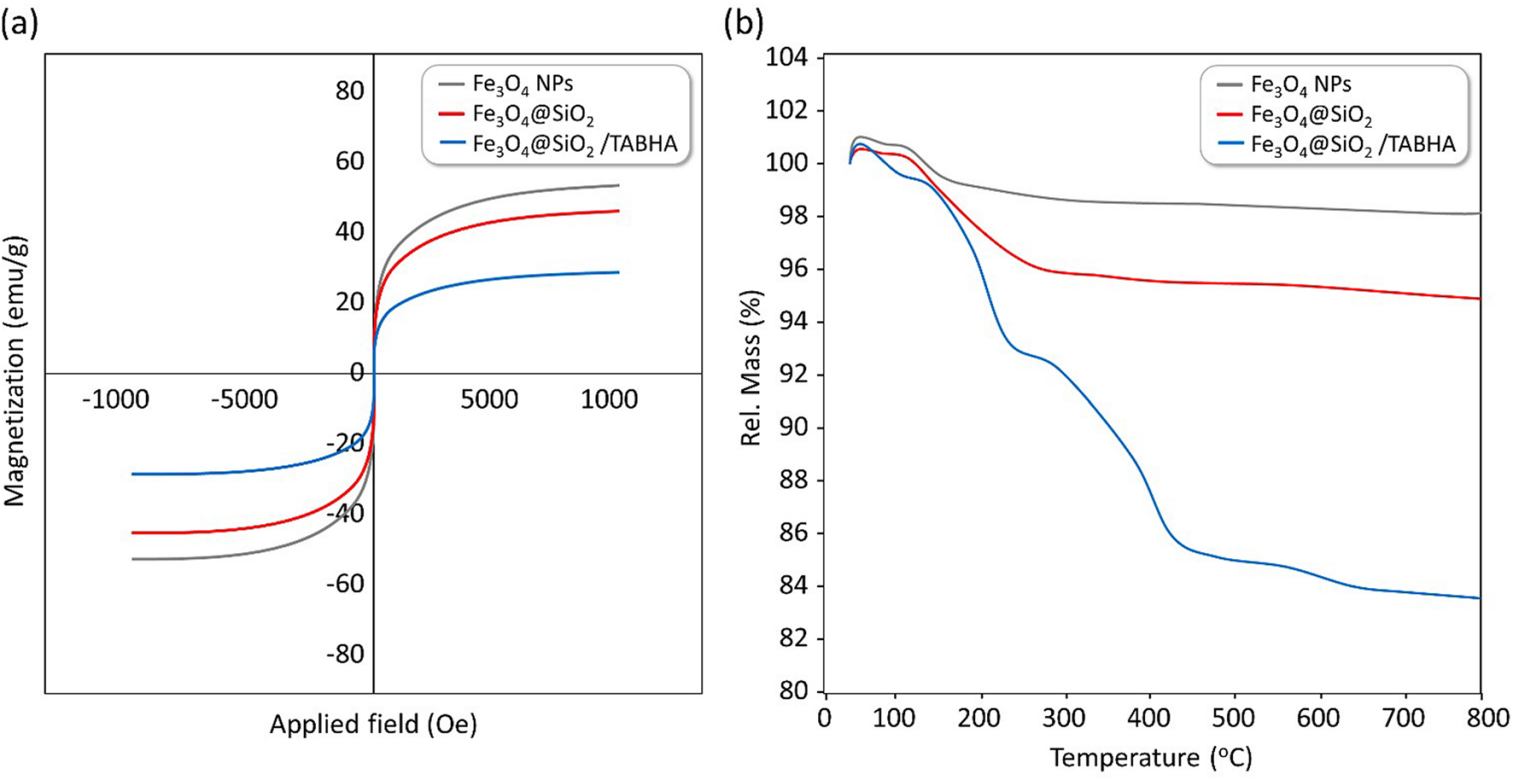
(**a**) VSM (room temperature), and (**b**) TGA (under air) curves of the neat Fe_3_O_4_, Fe_3_O_4_@SiO_2_, and Fe_3_O_4_@SiO_2_/TABHA NPs.

As one of the most important features of the prepared catalytic system, magnetic property is specially noticed because this feature is the main contributor to the convenient separation in the preparation and application stages. Due to the presence of the iron element in the core of this catalyst, it is possible to easily separate this catalyst from the reaction medium using an external catalyst, in comparison with other organocatalysts such as benzoisothiazolone or diselenide derivatives^58,59^. The results of vibrating-sample magnetometer (VSM) analysis on the samples of Fe_3_O_4_, Fe_3_O_4_@SiO_2,_ and Fe_3_O_4_@SiO_2_/TABHA NPs have been demonstrated in Fig. 5a, indicating super-paramagnetic behavior of the catalyst. Obviously, the magnetic feature is reduced proportional to coating of the core with more layers. More precisely, the magnetic property of Fe_3_O_4_ NPs is around 52 emu g^−1^, and it is reduced to around 42 emu g^−1^ after coating by silica layer, and more decreased to around 24 emu g^−1^ after coating with 2-thiazolyamine.

#### TGA analysis

The thermal resistance of the Fe_3_O_4_ NPs (grey curve), Fe_3_O_4_@SiO_2_ NPs (blue curve), and the fabricated Fe_3_O_4_@SiO_2_/TABHA nanoparticle (red curve) has been investigated by thermogravimetric analysis (TGA). As shown in the Fig. 5b, in all three samples, a slight increase in the weight is observed at the first stage. This increase for Fe_3_O_4_ is related to the physical absorption of the water on its surface, and for Fe_3_O_4_@SiO_2_ and Fe_3_O_4_@SiO_2_/TABHA NPs is related the entrapped water molecules into the silica network, which of course is more for Fe_3_O_4_@SiO_2_/TABHA NPs compared to Fe_3_O_4_@SiO_2_ NPs due to the presence of vinyl and 2-thiazolyamine. According to the literature, the organic layers present in the structure are removed through heating up to ca. 300 °C^60^. That is why, the samples have shown difference in a thermal range of 150–280 °C, and the percentage of mass reduction in Fe_3_O_4_@SiO_2_/TABHA NPs is higher than the other two samples. The observed difference in the weight loss is ascribed to the destruction of TABHA. After 280 °C, there is another decreasing shoulder in the blue curve, which can be attributed to degradation of vinyl groups. After that, from 420 °C onwards, the main destruction of the structure occurs. It is worth mentioning that due to the fact that there are no organic layers on Fe_3_O_4_@SiO_2_, its main destruction takes place in the earlier stages than the Fe_3_O_4_@SiO_2_/TABHA NPs.

#### XRD analysis

**Figure 6:**
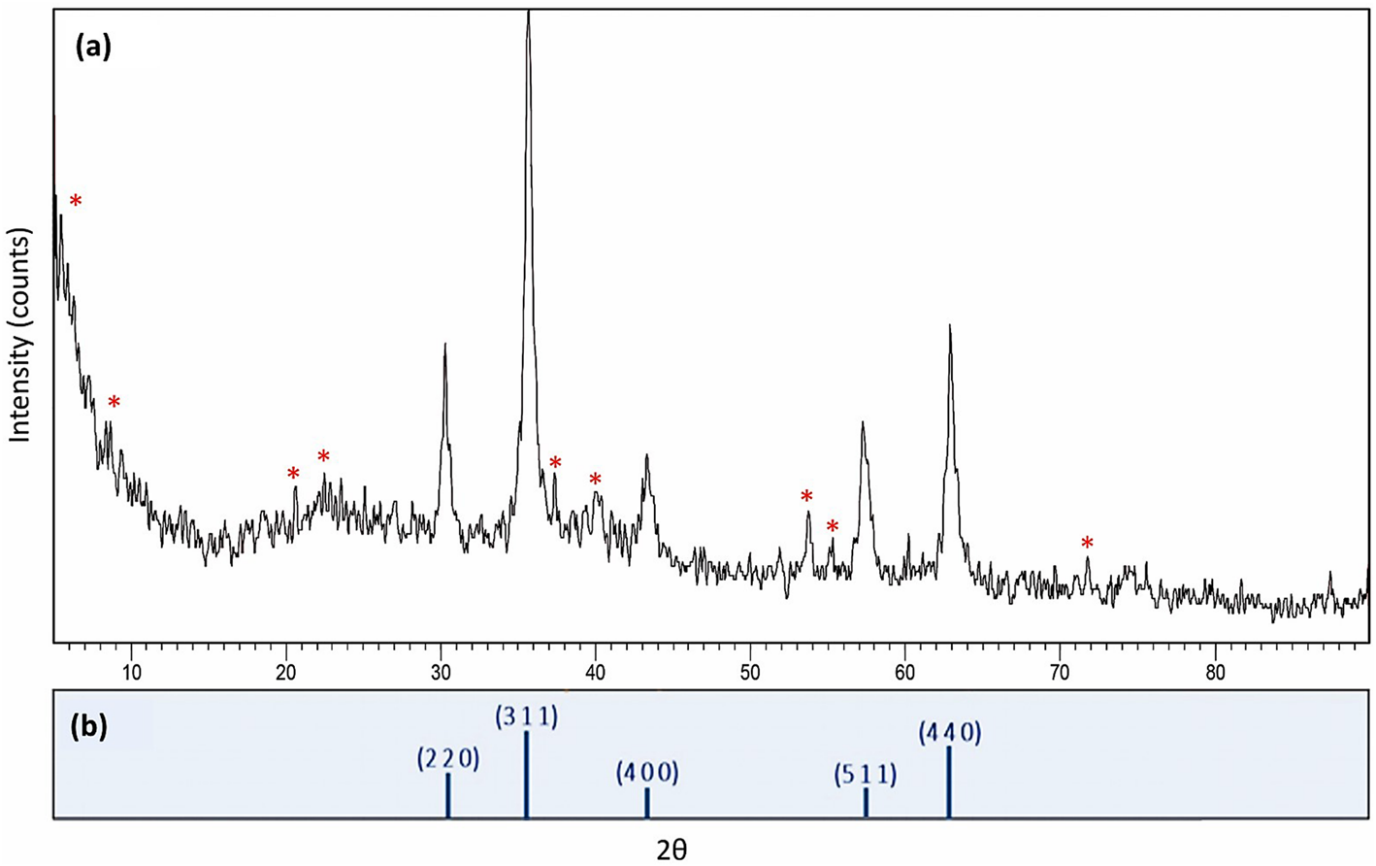
The XRD pattern of the fabricated Fe_3_O_4_@SiO_2_/TABHA NPs (**a**), and Fe_3_O_4_ MNPs (**b**).

The X-ray diffraction (XRD) pattern of the Fe_3_O_4_@SiO_2_/TABHA nanoparticle is shown in Fig. 6a. The peaks appeared at 2θ = 30.3°, 35.7°, 43.8°, 57.3° and 63.0° that are signed by Miller indices of (2 2 0), (3 1 1), (4 0 0), (5 1 1), and (4 4 0), respectively, are related to the XRD pattern of the Fe_3_O_4_ MNPs. The XRD pattern of Fe_3_O_4_ magnetic MNPs is obtained from *JCPDS* database (*PDF#99-0073*) (Fig. 6b)^61^. In the spectrum of the Fe_3_O_4_@SiO_2_/TABHA NPs, there are nine new peaks at 2θ = 6.3°, 8.5°, 20.69°, 22.74°, 37.52°, 40.32°, 53.58°, 55.34°, and 71.39°, which have been marked with red stars in the figure. Most likely, these new peaks are attributed to the new layer created through 2-thiazolyamine functionalization.

#### UV–vis diffuse reflectance spectroscopy

**Figure 7:**
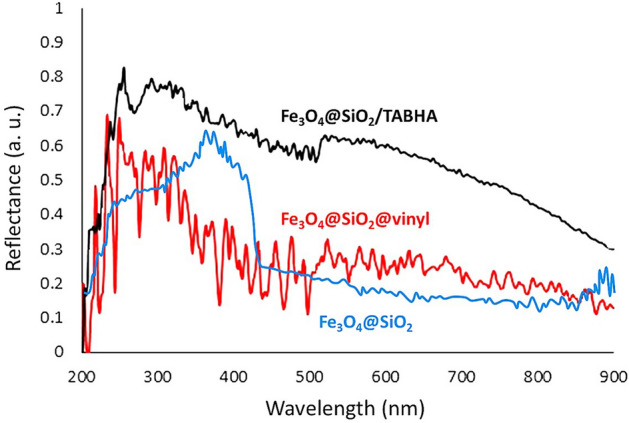
UV–DRS curves of Fe_3_O_4_@SiO_2_ (blue), Fe_3_O_4_@SiO_2_@vinyl (red), and Fe_3_O_4_@SiO_2_/TABHA NPs (black).

UV–vis diffuse reflectance spectroscopy (UV-DRS) measurements on the solid samples of Fe_3_O_4_@SiO_2_, Fe_3_O_4_@SiO_2_@vinyl, and Fe_3_O_4_@SiO_2_/TABHA NPs, was performed to investigate the optical activity and light-reflectance behavior of the Fe_3_O_4_@SiO_2_/TABHA NPs. As is observed in Fig. 7, the curves of all three samples exhibited different reflectance activity that can be a reason to claim that the structure of the final catalyst is different from Fe_3_O_4_@SiO_2_ and Fe_3_O_4_@SiO_2_@vinyl materials. The maximum reflectance of the Fe_3_O_4_@SiO_2_ NPs is in a range of 200–270 nm and also around 890 nm, while for the Fe_3_O_4_@SiO_2_@vinyl NPs, it is around 240–400 nm. For Fe_3_O_4_@SiO_2_/TABHA NPs a bread peak appeared at 200–900 nm, which confirms inclusion of more active ingredients in the structure.

### Catalytic application in peptide synthesis

So far, it has been explained how to synthesize our catalyst, Fe_3_O_4_@SiO_2_/TABHA NPs, and then, by examining various analyzes, it has been proved that the desired structure has been synthesized correctly. Also, by examining different conditions, the optimized condition for preparation of the catalyst was obtained. In the following, the performance of Fe_3_O_4_@SiO_2_/TABHA NPs in catalyzing the formation of peptide bonds is examined. The optimal conditions for the use of Fe_3_O_4_@SiO_2_/TABHA catalytic system in catalyzing the amidation reactions are presented through screen of the different conditions. The synthesized NPs were used to catalyze the formation of amide bonds between alanine and glycine, phenylalanine and glycine, cysteine and arginine to prove its ability to catalyze the formation of amide bond. It should be state that some of these amino acids were used in the protected from. The details of the experimental steps for the formation of an amide bond between the amino acids mentioned above are discussed in following, and its spectral information are available in supporting information (SI) (Figs. S2–S4).

#### Optimization of the catalytic process in peptide coupling reactions

In order to reach the optimized conditions for the use of the Fe_3_O_4_@SiO_2_/TABHA catalytic system, various amounts of the NPs, amount of TBTU as a conventional amide/peptide coupling reagent, P(OEt)_3_ as an additional molecular sieve were carefully monitored. The details of this investigation are reported in Table 2. It is observed that the catalyst amount and time directly affected the synthesis reaction of Fmoc-Ala-OH and glycine methyl ester with equal molar ratios (2.0 mmol). As is seen in Table 2, TBTU was also used as a coupling reagent, where 0.64 g of this reagent led to 76% yield, during 12 h, while Fe_3_O_4_@SiO_2_/TABHA NPs has performed the reaction much better than TBTU with a smaller amount and during less reaction time. It has been also revealed that the optimum conditions were obtained by using 0.2 g of the Fe_3_O_4_@SiO_2_/TABHA catalyst, in 4 h.

**Table 2:** Reaction optimization by utilizing different amounts of nanocatalyst.

Entry	Reagent	Condition	R.W^a^ (g)	Time (h)	Yield (%)
1	TBTU^b^	DIPEA/dry DCM, r.t./N_2_	0.64	12	76
2	Fe_3_O_4_@SiO_2_/TABHA	P(OEt)_3_/DCM, r.t./N_2_	0.2	4	85
3	Fe_3_O_4_@SiO_2_/TABHA	Dry DCM, r.t./N_2_	0.2	4	90^c^
4	Fe_3_O_4_@SiO_2_/TABHA	Dry DCM, r.t./N_2_	0.2	6	88
5	Fe_3_O_4_@SiO_2_/TABHA	Dry DCM, r.t./N_2_	0.15	8	84
6	Fe_3_O_4_@SiO_2_/TABHA	Dry DCM, r.t./N_2_	0.11	10	70

^a^Reagent weight.

^b^ *O*-(benzotriazol-1-yl)-N,N,N′,N′-tetramethyluronium tetrafluoroborate.

^c^ Optimum conditions.

In addition to the catalytic performance of the designed Fe_3_O_4_@SiO_2_/TABHA system, stereoselective function in the synthesis of diastereoisomers was also investigated. Actually, since the presented catalytic system does not include any chiral center, it cannot be expected for it to be able to induce diastereoselectivity within the peptide bond formation. To practically investigate this issue, Fmoc-l-Ala-l-Ala-COOMe and Fmoc-d-Ala-l-Ala-COOMe were synthesized in the presence of TBTU/HOBT (TBTU: 2-(1H-Benzotriazole-1-yl)-1,1,3,3-tetramethylaminium tetrafluoroborate, HOBT: Hydroxybenzotriazole). Then, the standard solutions of the prepared dipeptide structures were prepared and studied by RP-HPLC (Figs. S5 and S6, in the SI section). Afterward, the reaction was carried out in the presence of Fe_3_O_4_@SiO_2_/TABHA catalytic system, and the RP-HPLC spectra of the synthesized dipeptide structure was provided and compared with the reference spectra (Fig. S7). As is observed and it was expected, the chirality is not retained by the prepared Fe_3_O_4_@SiO_2_/TABHA catalytic system, and a mixture of Fmoc-l-Ala-l-Ala-COOMe and Fmoc-d-Ala-l-Ala-COOMe was obtained. In order to retain the chirality and induce selective synthesis of diastereoisomers, HOBT may be needed to be used along with the Fe_3_O_4_@SiO_2_/TABHA particles^62^. In this regard, the activity of the designed catalyst in the presence of HOBT was experimented. Concisely, it was observed that the chirality is largely retained by the use of Fe_3_O_4_@SiO_2_/TABHA/HOBT (Fig. S8). The experimental procedure related to this experiment has been given in the SI section.

#### Catalyst recyclability

**Figure 8:**
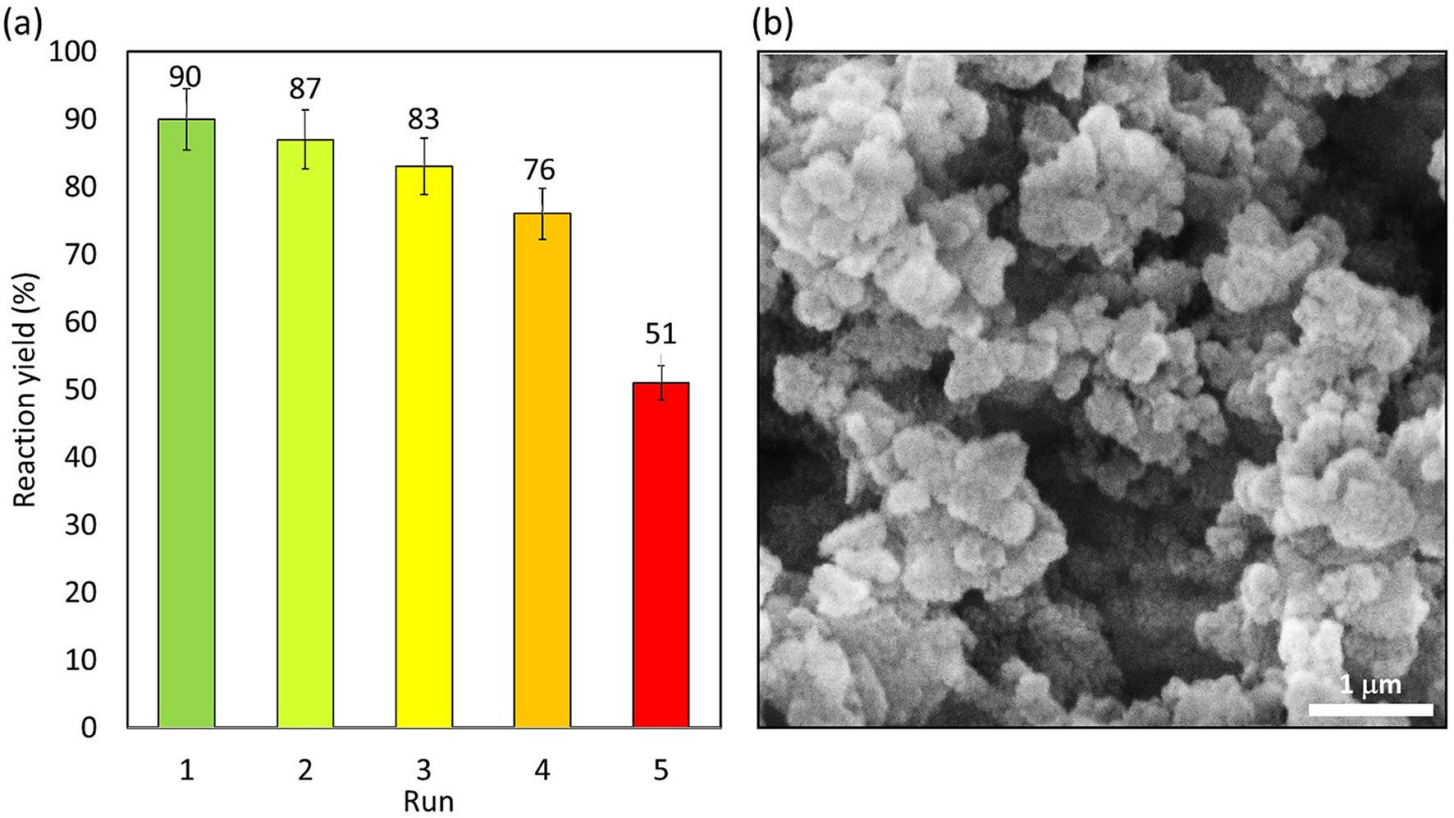
(**a**) Recyclability investigation of Fe_3_O_4_@SiO_2_/TABHA NPs in catalyzed peptide coupling reactions. The results were obtained from the coupling reaction between Fmoc-Phe-OH and methyl glycinate, per 0.2 g of the catalyst at room temperature. (**b**) SEM image of recovered Fe_3_O_4_@SiO_2_/TABHA MNPs after five times recycling.

In order to evaluate the reusability of the prepared Fe_3_O_4_@SiO_2_/TABHA catalytic system, the NPs were magnetically collected from the reaction mixture after completion of the reaction, and prepared for further cycles. The collected particles were washed several times with distilled water and dried in an oven. The Fe_3_O_4_@SiO_2_/TABHA NPs were used for five successive times in the model reaction, which is the peptide coupling reaction between Fmoc-Phe-OH and glycine methyl ester. As shown in the Fig. 8a**,** monitoring the catalytic process confirms that the reaction yield has not changed significantly, so that after a four-time recovering, less than 15% of the reaction yield was reduced. According to the Fig. 8b, which is SEM image of the recovered Fe_3_O_4_@SiO_2_/TABHA NPs, after five times recycling, Fe_3_O_4_@SiO_2_/TABHA are aggregated and do not have a spherical shape with a mono-dispersed pattern. Possibly, the observed decrease in the catalytic activity is due to these structural changes that lead to decreased active surface area of in the Fe_3_O_4_@SiO_2_/TABHA NPs. As is observed in the provided images (Fig. S9, in the SI section), the heterogeneous particles of Fe_3_O_4_@SiO_2_/TABHA catalyst are stable in the solution medium (image a). They, are participated via holding an external magnet at the bottom of the flask (image b). Generally, oxidation of the Fe_3_O_4_ NPs and the amine groups located onto the surfaces by O_2_ (air) causes change in the color from very dark (image c) to light brown after the time (image d). Oxidation of Fe_3_O_4_ NPs may result in conversion to Fe_2_O_3_ NPs that are less magnetic^63^. In this state, the catalytic performance of the particles is in part lost. To elongate shelf-time of the prepared Fe_3_O_4_@SiO_2_/TABHA NPs, N_2_ gas is merged into the vial that is well sealed via phenolic cap and parafilm, and stored at 4 °C in refrigerator.

#### Suggested mechanism

**Figure 9:**
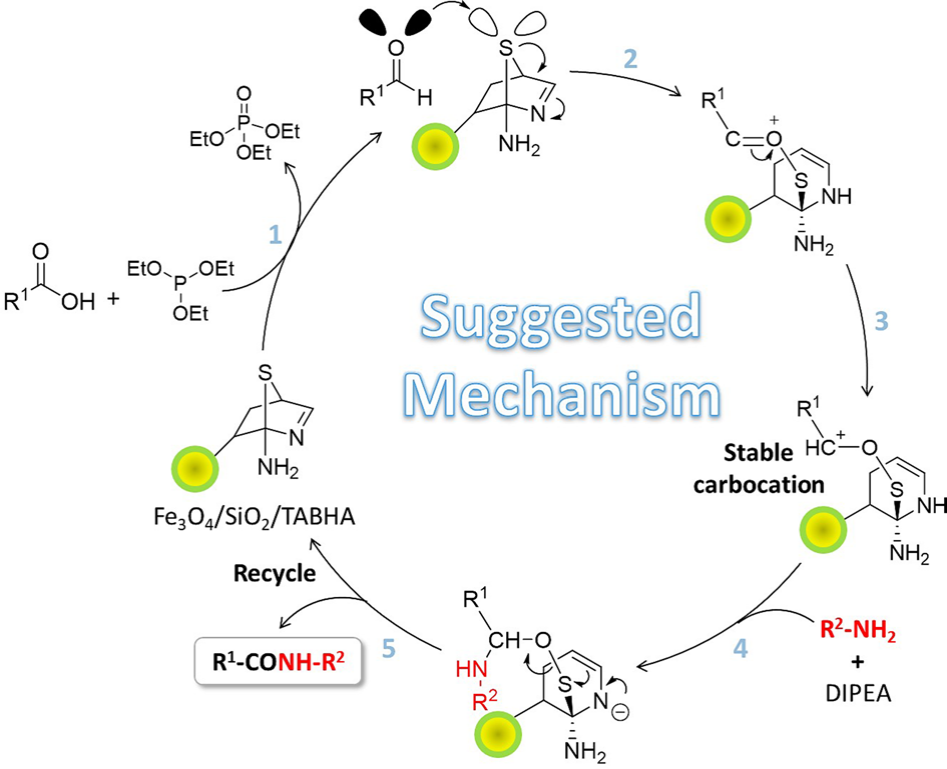
A plausible multi-stage mechanism for the synthesis of the dipeptides by the fabricated Fe_3_O_4_@SiO_2_/TABHA catalytic system.

A plausible mechanism for the amide/peptide bond formation by the prepared Fe_3_O_4_@SiO_2_/TABHA catalytic system is shown in the Fig. 9. The process of this mechanism is occurred through addition of N-protected amino acids, and then the Fe_3_O_4_@SiO_2_/TABHA NPs is recycled during the reaction. The first stage of this mechanism is started with the use of triethylphosphite as an initial reducing agent that reduces amino acids. It should be noted that the protected amino acids should be in their canonical state (non-protonated state) via controlling the pH (isoelectric point). In the next step, a nucleophilic attack by the reduced amino acid is performed on the sulfur atom of the catalyst, and thus, by breaking of the S–C bond, one of the catalyst rings opens and a positive charge is created on the oxygen atom. Oxygen has high electronegativity, so the compound that contains a positively charged oxygen atom is often unstable. This is why in the third stage, the electron pair between the positively charged oxygen and carbon are placed on positively charged oxygen, resulting in the formation of a stable carbocation. Due to the formation of this carbocation, it is necessary to use a dry solvent and a neutral atmosphere as reaction conditions. The fourth stage involves a nucleophilic attack by the amine group of glycine methyl ester to the carbocation formed in the third stage. In the fifth step, as the last step of this proposed mechanism, 2-aminothiazole ring is closed, and an amide/peptide bond is formed^31,58^.

#### Comparison of Fe_3_O_4_@SiO_2_/TABHA catalytic process with solid-phase method

In order to highlight the advantages of the presented Fe_3_O_4_@SiO_2_/TABHA catalytic system, a brief comparison with the solid-phase peptide synthesis (SPPS) method was made. Generally, to have a meaningful comparison, the most important factors such as reaction time, yield, purity, complexity, required additive compounds, and cost were considered. For this comparison, the synthetic reaction of Fmoc-l-Ala-l-Ala-COOMe dipeptide was considered as a model reaction, and the provided RP-HPLC spectra (reported as Fig. S8, in the SI section) were considered. According to Table 3, the time of the catalytic process of Fe_3_O_4_@SiO_2_/TABHA is equal the SPPS method (4 h). In fact, 2 h out of four is dedicated to washing and swelling of the CTC (CTC stands for 2-chlorotrityl chloride) resin. There was no significant difference between the reaction yields obtained via two different methods, while the purity value of ca. 98% was obtained by the Fe_3_O_4_@SiO_2_/TABHA catalyst. This value was obtained ca. 94% in the SPPS method. As another determinative factor, convenience of the method is seriously considered by the researchers. According to the SPPS principles, several successive stages should be passed, at which large volumes of the solvents are consumed. Whereas, a single-stage process is executed by the prepared Fe_3_O_4_@SiO_2_/TABHA catalyst. Moreover, TBTU as an amide/peptide coupling reagent, and diisopropylethyl amine (DIEA) are required in the SPPS method, which are relatively expensive reagents in comparison with triethylphosphite. Due to consuming large volumes of DMF and DCM solvents, and also high prices of CTC resin and coupling reagents, SPPS is known as an expensive synthetic method. As another advantages of the presented catalytic method, reusability of the Fe_3_O_4_@SiO_2_/TABHA catalyst that was discussed in section “Catalyst recyclability” of this paper, can be referred as well. While, no component in the SPPS strategy is recyclable.

**Table 3:** Comparative information of Fmoc-l-Ala-l-Ala-COOMe dipeptide synthesis by the designed Fe_3_O_4_@SiO_2_/TABHA catalytic system, and solid-phase peptide synthesis method.

Parameter	Fe_3_O_4_@SiO_2_/TABHA method	SPPS method
Process time (min)	4 h	4 h
Yield (%)	90	94
Purity (%)	> 98.0	94.4
Complexity	Low	High
Additive materials	Triethylphosphite	TBTU, DIEA
Cost	Low	High
Reusability	Four cycles	No recycle

## Experimental section

### Materials and equipment

All the chemicals, reagents, and equipment used in this study are listed in the Table 4.

**Table 4:** Chemicals and equipment used in this study.

Materials and equipment	Purity and brand
FeCl_2_·4H_2_O	Sigma Aldrich (98%)
FeCl_3_·6H_2_O	Sigma Aldrich (≥ 98%)
Ammonia	Merck (25%)
Ethanol	Sigma Aldrich (97%)
TEOS	Sigma Aldrich (98%)
Trimethoxyvinylsilane (TMVS)	Sigma Aldrich (97%)
Dimethylformamide (DMF)	Sigma Aldrich (99.8%)
PdCl_2_	Nanoshel (99.9%)
PPh_3_	Sigma Aldrich (99%)
HCl	Merck (37%)
AgNO_3_	Indiamart—99.9%
NaoAc	Sigma Aldrich (≥ 99%)
2-Aminothiazole	Sigma Aldrich—97%
Triethylphosphite	Sigma Aldrich – 98%
HOBT	Sigma Aldrich
FTIR analysis	Shimadzu IR-470 spectrometer
EDX analysis	Numerix DXP–X10P
SEM analysis	Sigma-Zeiss, microscope
TEM analysis	Philips Cm 12 Instrument
VSM analysis	Lakeshore 7407
TGA analysis	STA504 device
XRD analysis	JEOL JDX–8030 (30 kV, 20 mA)
HPLC	Agilent Technologies, Santa Clara
DLS	Horiba (SZ-100)
UV–vis diffuse reflectance spectroscopy	UV-1280 Shimadzu
Ultrasound cleaning bath	KQ-250 DE (40 kHz, 250 W)

### Preparation methods

#### Preparation of Fe_3_O_4_ MNPs

In order to produce Fe_3_O_4_ MNPs, initially, 3.0 g of FeCl_3_.6H_2_O and 1.5 g of FeCl_2_.4H_2_O were dissolved in 100.0 mL of deionized water in a 250.0 mL round-bottom flask, and the content was stirred at 70 °C, under N_2_ atmosphere. The temperature was then reduced to 50 °C and 10 mL of ammonia 25% was added dropwise during 75 min, resulting in the formation of a dark particles. After cooling down the reaction mixture to room temperature, the dark magnetic NPs were separated with an external magnet and washed for several times with ethanol, and then dried in oven at 70 °C.

#### Preparation of the core/shell Fe_3_O_4_@SiO_2_ MNPs

In a round-bottom flask (100 mL), 0.225 g of the prepared F_3_O_4_ was dispersed in 25 mL of deionized water by using an ultrasonic bath, for 15 min. Then, while stirring, 7.5 mL of ammonia 25% was added dropwise to the mixture. Then, 80 mL of ethanol with high purity was added to the mixture, and after 10 min, 4.0 mL of TEOS was added to the mixture. After completion of the addition, the mixture was stirred under reflux condition, for 24 h. The resulting product was collected by magnetic separation and washed with ethanol.

#### Preparation of Fe_3_O_4_@SiO_2_@vinyl MNPs

First, 10.0 g of the prepared Fe_3_O_4_@SiO_2_ was placed into a three-necked flask (100 mL) containing 70 mL of toluene. Then, 3.54 g of trimethoxy vinylsilane (TMVS) was added dropwise to the reaction mixture over 10 min, at room temperature. Next, the mixture was stirred for 24 h under reflux conditions in chloroform (7.0 mL). Finally, Fe_3_O_4_@SiO_2_@vinyl NPs were collected by an external magnet and washed with ethanol several times.

#### Preparation of Fe_3_O_4_@SiO_2_/TABHA NPs

Mixture A: 1.0 g of Fe_3_O_4_@SiO_2_@vinyl NPs containing and 7.0 mL of DMF was placed in a round-bottom flask (25 mL), and the resulting mixture was completely dispersed using ultrasonic for 10 min. Mixture B: in a beaker, 0.0354 mg of PdCl_2_ was dissolved in 10 mL of HCl (0.5 M) by stirring at 60 °C, for 2 h. Then, mixture B was added to mixture A, and the resulting mixture was magnetically stirred for 2 h at room temperature. In the next step, 0.1 mL of triethylamine (TEA), 0.01 g of NaOAc, 0.05 g AgNO_3_, and 0.2 g of 2-aminothiazole were added to the mixture, and the contents were stirred under reflux condition, for 24 h. Finally, Fe_3_O_4_@SiO_2_/TABHA NPs were collected by an external magnet and washed with ethanol several times.

#### General procedure for the synthesis of dipeptide with the catalytic system of Fe_3_O_4_@SiO_2_/TABHA

Initially, Fe_3_O_4_@SiO_2_/TABHA NPs (0.05 g) were dispersed in dry DCM (5.0 mL) using an ultrasound bath (50 kHz, 100 W L^−1^), for an adequate time. Then, triethylphosphite (53.2 μL, 0.310 mmol) and 2.0 mmol of the N-protected amino acid were added to the flask and the resulting mixture was stirred for 30 min, under a N_2_ atmosphere. Next, 2.0 mmol of acid-protected amino acids was added and the mixture was stirred for 3 h, under N_2_ atmosphere at room temperature. After completion of the reaction, the magnetic NPs were separated from the reaction mixture by an external magnet, washed with methanol, and then dried in an oven at 60 °C. The progress of the reaction was frequently monitored by thin-layer chromatography (TLC). The extraction process was performed by adding excess dry DCM to the mixture. Then, the DCM phase was evaporated by a rotary evaporator. The desired product (and a small amount of triethylphosphate impurity) were obtained as a powder and dried at room temperature. The synthesized dipeptide compounds were identified by H-NMR spectroscopy, given in the SI section.

## Conclusion

Today, protein–drug conjugates as the next generation of the pharmaceutical compounds have attracted huge attentions of researches. In this regard, design and preparation of the novel and more efficient coupling reagents that can be easily separated from the reaction mixture and recycled has prospered. In this study, a novel nanoscale peptide coupling reagent has been presented that demonstrated great potential to be utilized in the peptide bond formation reactions. The prepared coupling reagent well assisted the peptide bond formation resulting in ca. 90% reaction yield during 4 h, under mild conditions. The construction of the presented catalytic system was performed based on iron oxide MNPs. Then, the surface of the MNPs has been modified by the silane compounds, and then functionalized with 2-aminothiazole via Diels–Alder approach. FT-IR spectroscopy, SEM, TEM, EDX spectroscopy, XRD spectroscopy, TGA, VSM, and UV–vis DRS analyzes were used to characterize the catalytic structure and application of the synthesized nanoparticle. As the most important feature, the designed catalyst was easily separated from the reaction medium by an external magnet, which has helped the catalyst take an important step towards approaching green chemistry. Due to showing high structural properties such as super-paramagnetic property, thermal stability, and recyclability, and also significant catalytic performance in the peptide bond formation reactions, the presented catalytic system (formulated as Fe_3_O_4_@SiO_2_/TABHA) is recommended for scaling up and industrial applications.

## Supplementary Information

Supplementary Information.
