# Supplementary material for: Convenient synthesis of dipeptide structures in solution phase assisted by a thioaza functionalized magnetic nanocatalyst
Source: Sci Rep. 2022 Mar 18;12:4719. doi: 10.1038/s41598-022-07303-3 (PMC8933478; doi:10.1038/s41598-022-07303-3)
Supplement: Supplementary file 1 — Supplementary Information. [file 41598_2022_7303_MOESM1_ESM.docx]

***Supporting Information***

**Convenient Synthesis of Dipeptide Structures in Solution Phase Assisted by a Thioaza Functionalized Magnetic Nanocatalyst**

*Reza Taheri-Ledari, Fereshteh Rasouli Asl, Mahdi Saeidirad, Amir Kashtiaray, Ali Maleki**

*Catalysts and Organic Synthesis Research Laboratory, Department of Chemistry, Iran University of Science and Technology, Tehran 16846-13114, Iran.*

**Corresponding author. E-mail: maleki@iust.ac.ir; Fax: +98-21-73021584; Tel: +98-21-73228313.*


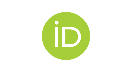
*Author’s ORCIDs:*

*Reza Taheri-Ledari: https://orcid.org/0000-0002-6511-9411*

*Ali Maleki: https://orcid.org/0000-0001-5490-3350*

***Table of Content***

| Content | Page |
| --- | --- |
| **Figure S1**. Intensity-based DLS curve of Fe_3_O_4_@SiO_2_/TABHA nanoparticles | S2 |
| **Figure S2**. H-NMR Spectra and spectral data of Fmoc-Ala-Gly-OMe dipeptide structure. | S3 |
| **Figure S3**. H-NMR Spectra and spectral data of Fmoc-Phe-Gly-OMe dipeptide structure. | S4 |
| **Figure S4**. H-NMR Spectra and spectral data of Cys-Arg dipeptide structure. | S5 |
| **Figure S5**. RP-HPLC result of Fmoc-L-Ala-L-Ala-COOMe, synthesized by TBTU/HOBT. | S6 |
| **Figure S6**. RP-HPLC result of Fmoc-D-Ala-L-Ala-COOMe, synthesized by TBTU/HOBT. | S7 |
| **Figure S7**. RP-HPLC result of Fmoc-L-Ala-L-Ala-COOMe, synthesized by Fe_3_O_4_@SiO_2_/TABHA catalytic system. | S8 |
| **Figure S8**. RP-HPLC result of Fmoc-L-Ala-L-Ala-COOMe, synthesized by Fe_3_O_4_@SiO_2_/TABHA catalytic system in the presence of HOBT. | S9 |
| **Figure S9**. Digital images provided from fresh sample of Fe_3_O_4_@SiO_2_/TABHA particles in solution (a,b) and dried (c), and oxidized sample via exposure to the air (d). | S10 |

**
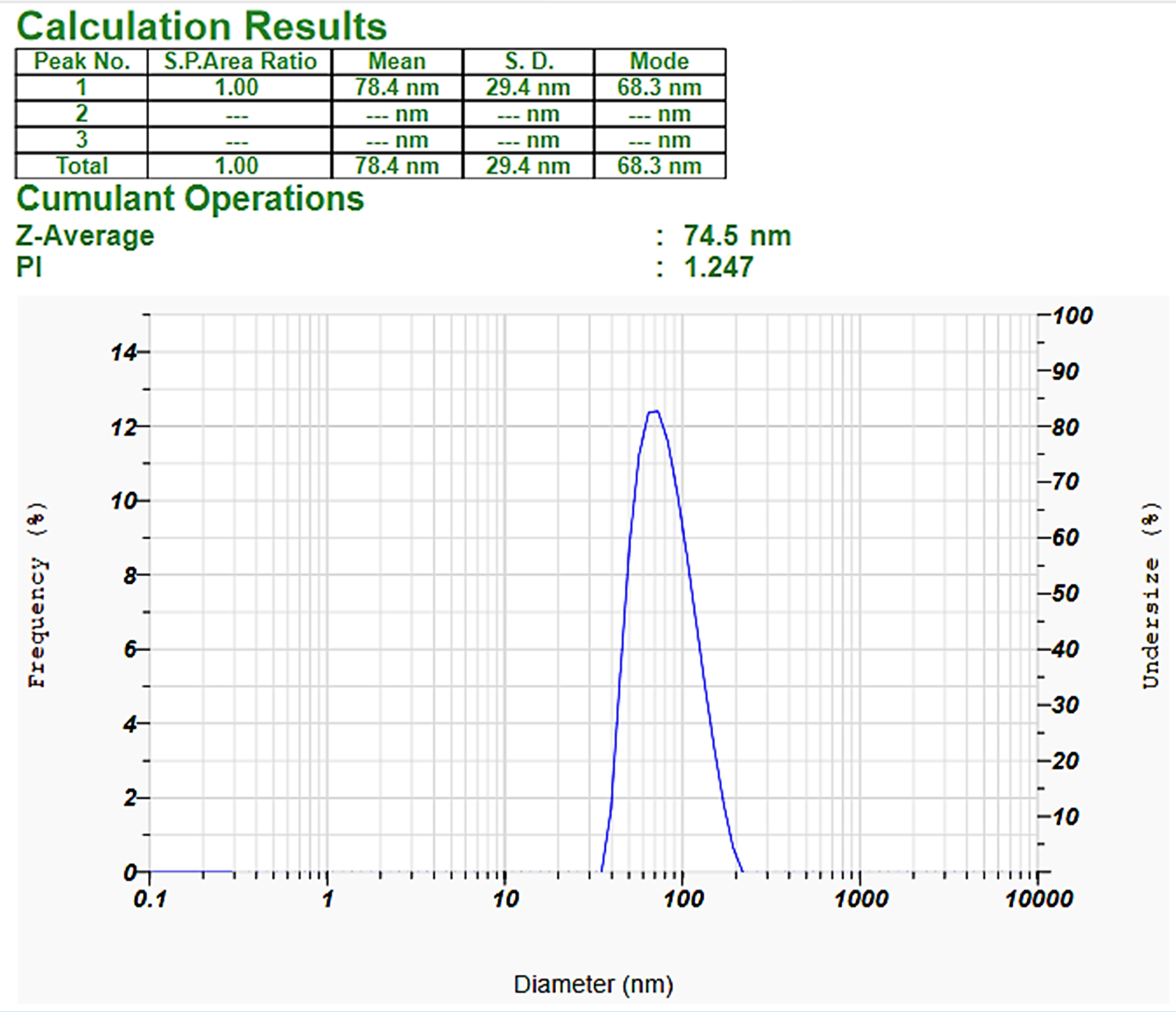
**

**Figure S1**. Intensity-based DLS curve of Fe_3_O_4_@SiO_2_/TABHA NPs.

**H-NMR Spectra and spectral data**

***Fmoc-Ala-Gly-OMe***

^1^H NMR (500 MHz, DMSO): δ = 1.26 (d, 3H, J = 7 Hz, CHC*H_3_*), 3.64 (s, 3H, COOC*H_3_*), 3.80-3.95 (qd, 1H, J = 16.5 Hz, J = 6.5 Hz, NHC*H*CH_3_), 4.12 (t, 1H, J = 6.5 Hz, C*H*CH_2_O), 4.23 (d, 2H, J = 6.5 Hz, CHC*H_2_*O), 4.27 (s, 2H, NHC*H_2_*CO), 7.34 (t, 2H, J = 7 Hz, Ar), 7.43 (t, 2H, J = 7 Hz, Ar), 7.58 (d, 1H, J = 7.5 Hz, OCON*H*CH), 7.73-7.76 (t, 2H, J = 7.5 Hz, Ar), 7.90 (d, 2H, J = 7.5 Hz, Ar), 8.31 (s, 1H, CON*H*CH_2_).


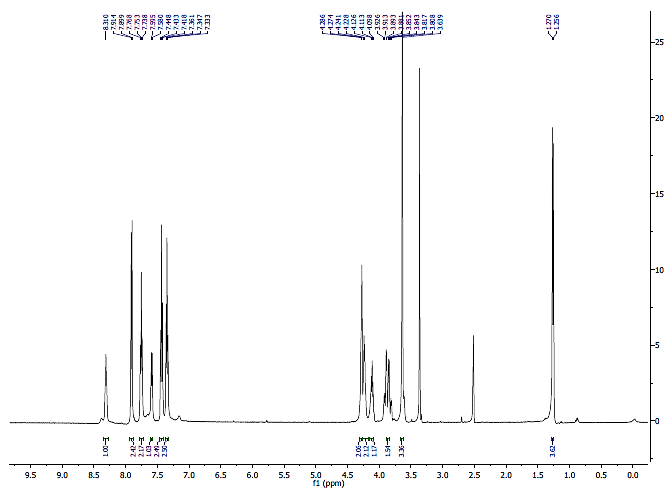


**Figure S2**. H-NMR Spectra and spectral data of Fmoc-Ala-Gly-OMe dipeptide structure.

***Fmoc-Phe-Gly-OMe***

^1^H NMR (500 MHz, DMSO): δ = 2.81 (t, 1H, J = 11 Hz, PhC*H_2_*), 3.06 (d, 1H, J = 15 Hz, PhC*H_2_*), 3.65 (s, 3H, COOC*H_3_*), 3.89-3.93 (m, 2H, NHC*H_2_*COOCH_3_), 4.10-4.18 (m, 3H, C*H*C*H_2_*OCONH), 4.29-4.34 (td, 1H, J = 3.5 Hz, J = 12.5 Hz, NHC*H*CH2Ph), 7.18-7.2 (t, 1H, J = 5, CON*H*CH_2_COOCH_3_), 7.25-7.35 (m, 7H, Ar), 7.39-7.43 (m, 2H, Ar), 7.63-7.71 (m, 2H, Ar), 7.88 (d, 2H, J = 7.5 Hz, Ar), 8.54 (d, 1H, J = 5.5 Hz, OCON*H*CH).

**
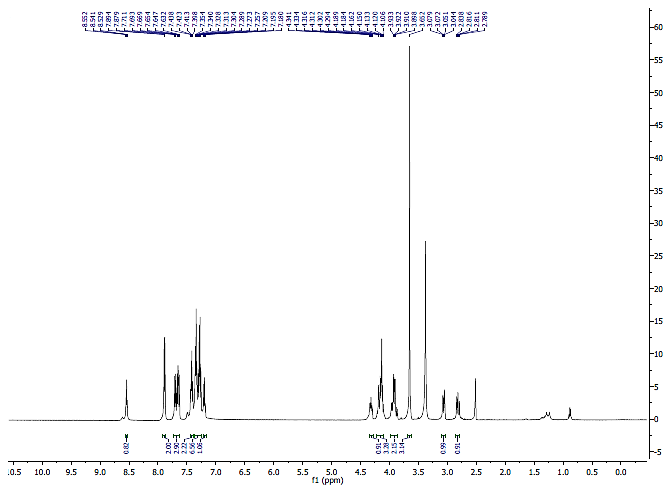
**

**Figure S3**. H-NMR Spectra and spectral data of Fmoc-Phe-Gly-OMe dipeptide structure.

**Cys-Arg**

^1^H NMR (500 MHz, DMSO): δ = 1.58-1.66 (m, 2H, C*H2*), 1.73-1.83 (m, 2H, C*H2*), 2.02 (t, 2H, *J*= 10 Hz, C*H2*), 2.79 (s, 1H, SH), 3.01-3.17 (m, 2H, CH2), 3.86 (t, 1H, J = 10 Hz, CH), 5.10 (t, 1H, *J*= 10.5 Hz, CH), 6.28 (s, 4H, NH_2_), 7.71 (s, 2H, NH_2_), 8.06 (s, 1H, N*H*).


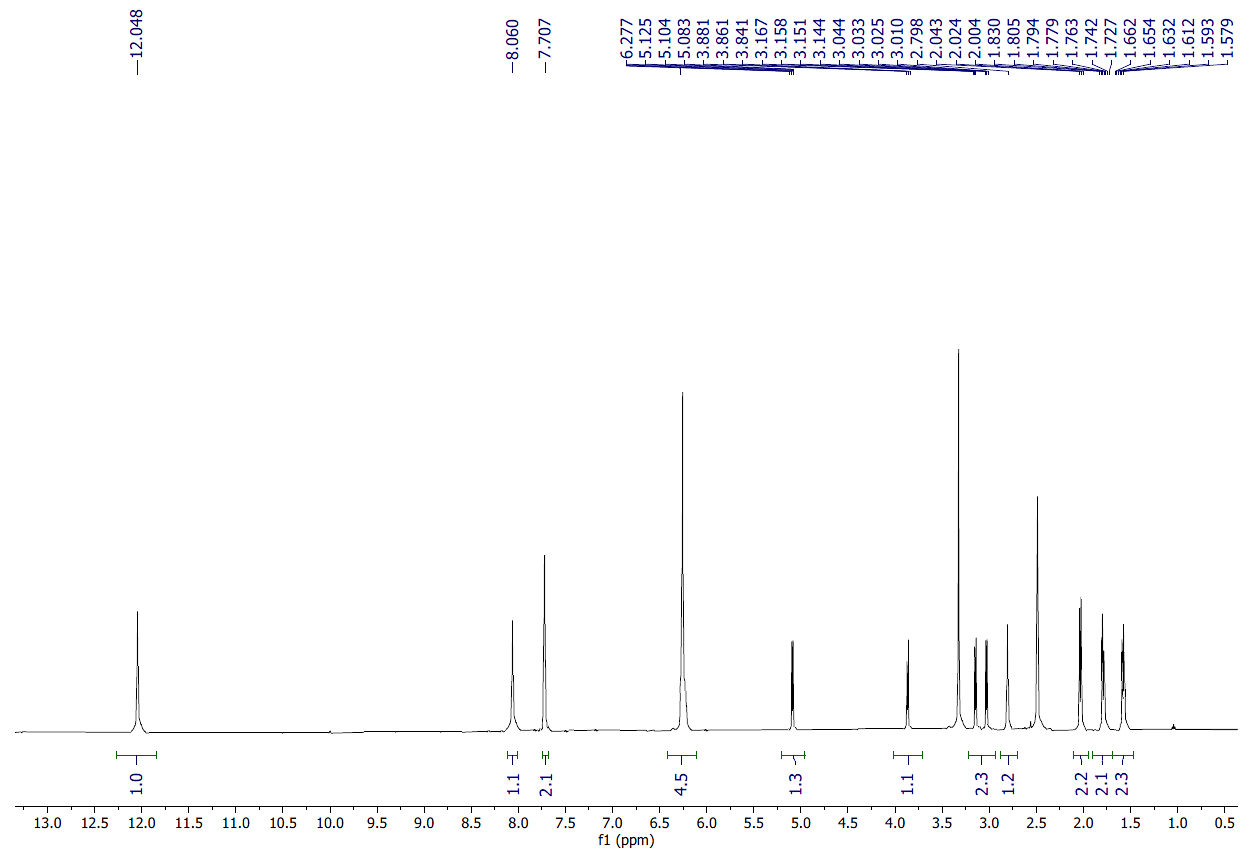


**Figure S4**. ^1^H NMR spectrum for compound Cys-Arg.


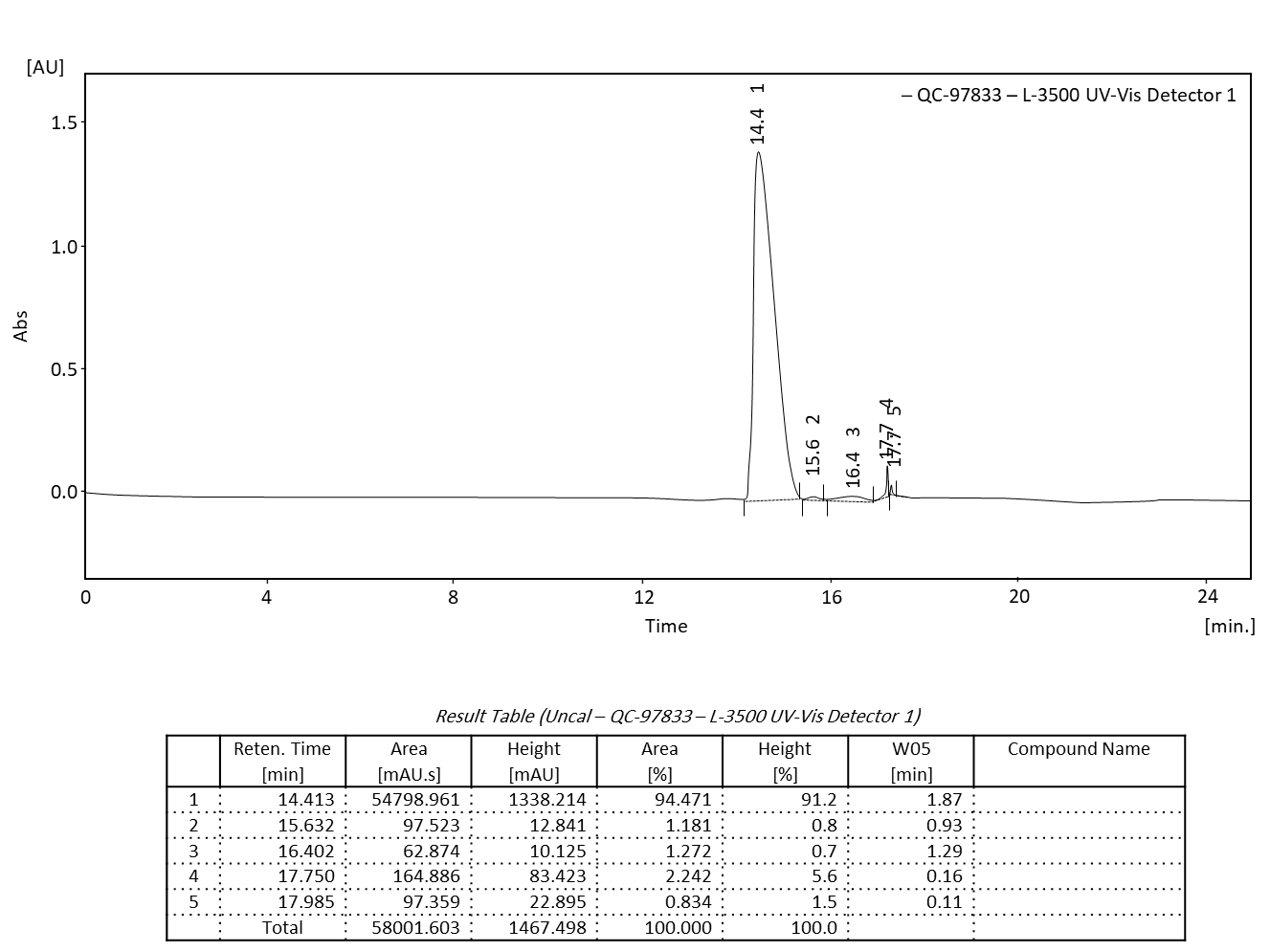


**Figure S5**. RP-HPLC result of Fmoc-L-Ala-L-Ala-COOMe, synthesized by TBTU/HOBT.

**HPLC conditions**

The HPLC analysis was performed using an Agilent 1200 system equipped with a quaternary pump, a degasser, an autosampler, a thermostated column compartment, and a variable wavelength detector. A reversed-phase (RP) column NUCLEODUR Sphinx (4.6 mm ID × 250 mm) connected with a C18 security guard column (3.0 mm ID × 4 mm) was used. The mobile phase consisted of MeOH and 20 mM sodium phosphate buffer (pH 5.5).


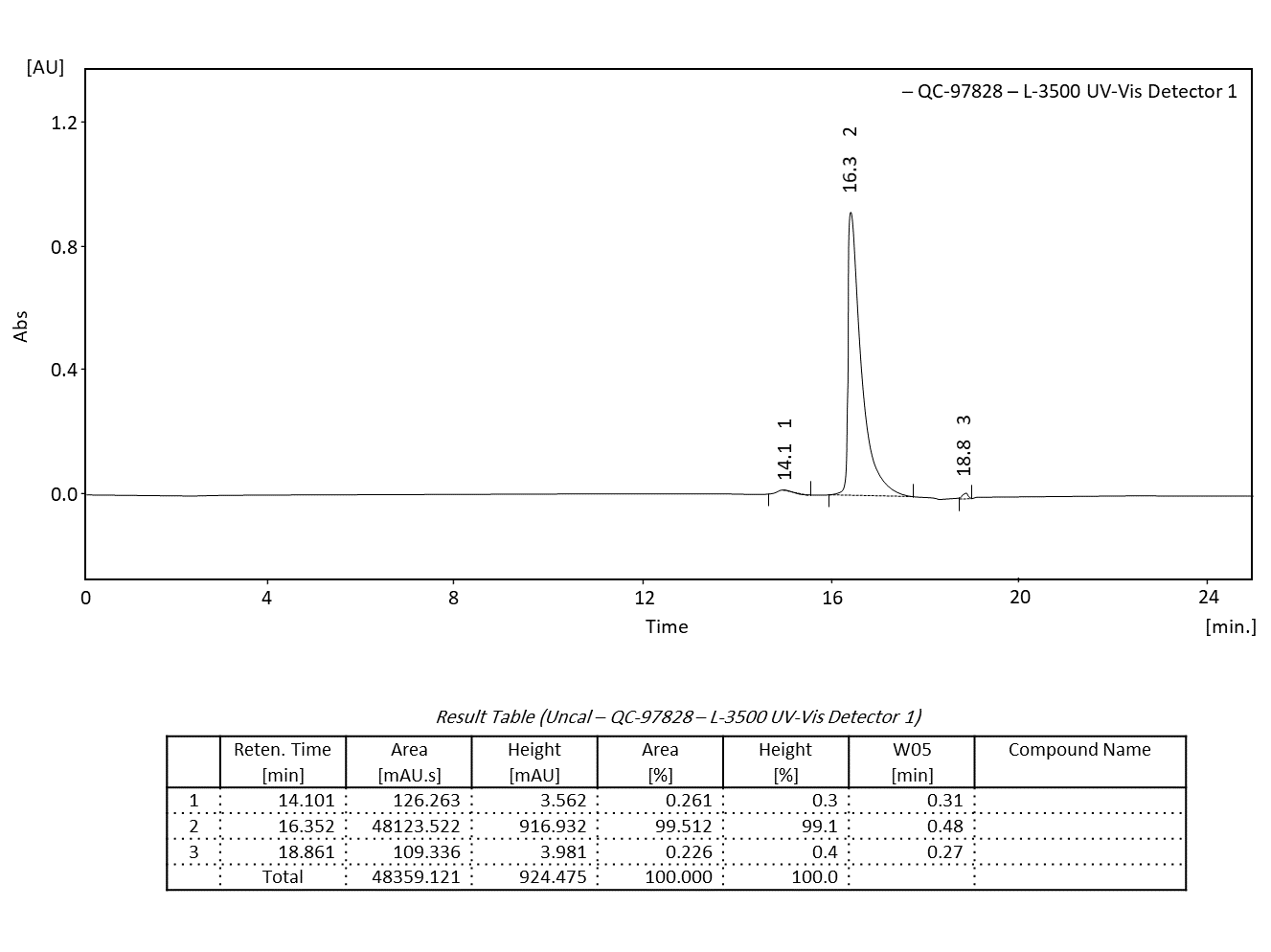


**Figure S6**. RP-HPLC result of Fmoc-D-Ala-L-Ala-COOMe, synthesized by TBTU/HOBT.


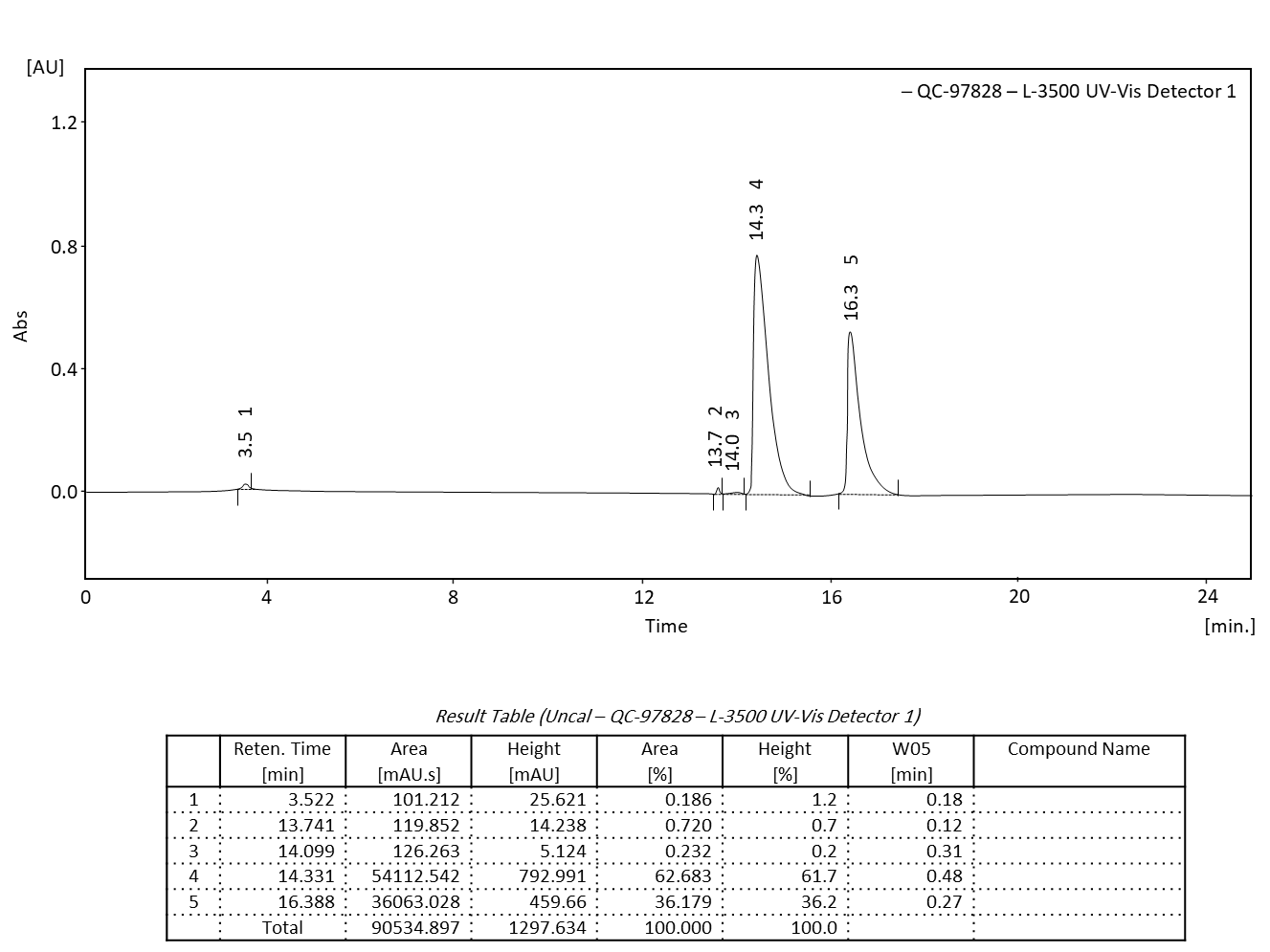


**Figure S7**. RP-HPLC result of Fmoc-L-Ala-L-Ala-COOMe, synthesized by Fe_3_O_4_@SiO_2_/TABHA catalytic system.


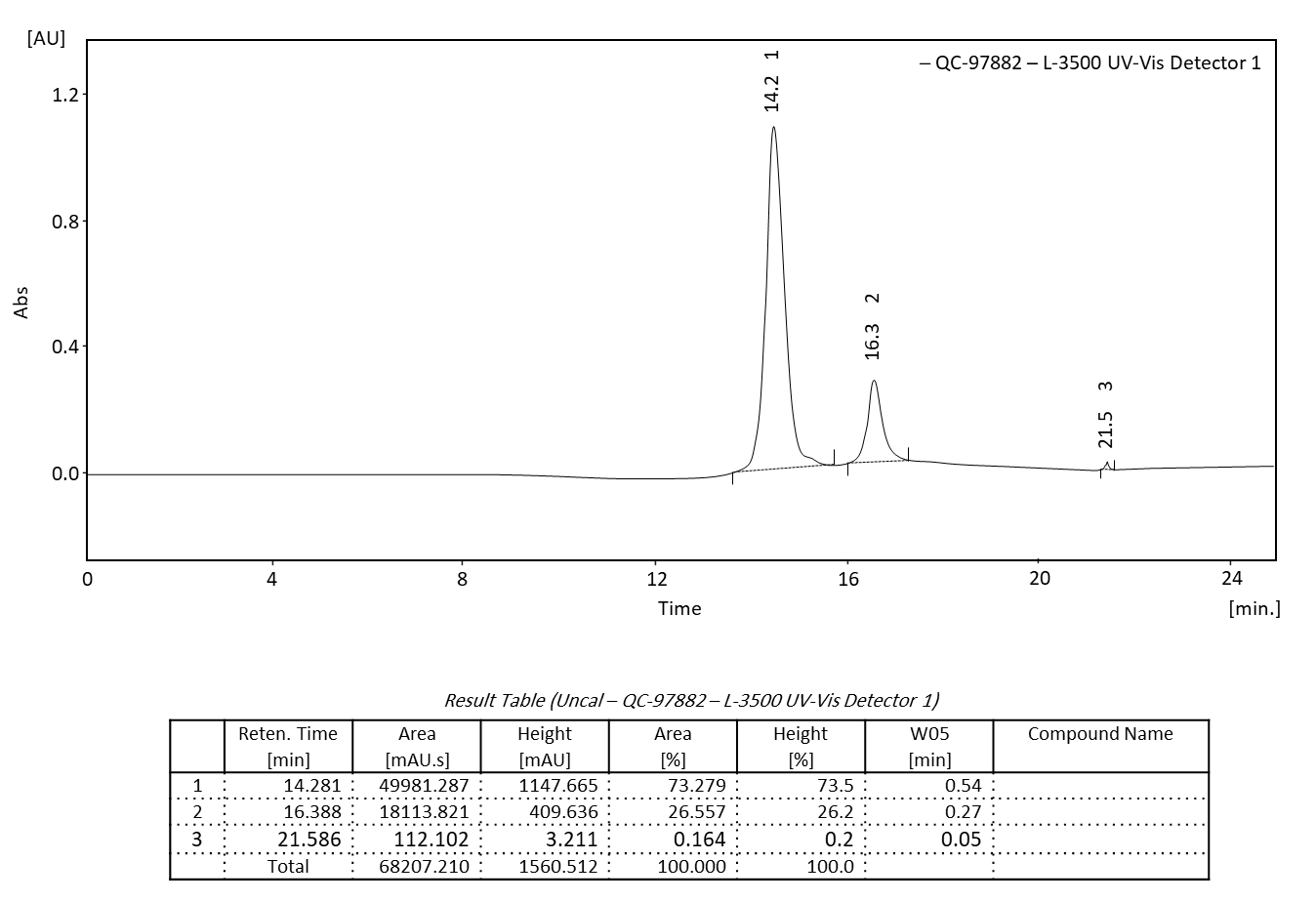


**Figure S8**. RP-HPLC result of Fmoc-L-Ala-L-Ala-COOMe, synthesized by Fe_3_O_4_@SiO_2_/TABHA catalytic system in the presence of HOBT.

**Experimental procedure**

Fe_3_O_4_@SiO_2_/TABHA particles (0.05 g) were dispersed in dry DCM (5.0 mL) using an ultrasound bath (50 KHz, 100 W L^-1^). Then, triethylphosphite (53.2 μl, 0.310 mmol), 2.0 mmol of the Fmoc-L-Ala-OH, and 2.0 mmol of HOBT (hydroxybenzotriazole) were added to the flask and stirred for 30 min under a N_2_ atmosphere. Next, 2.0 mmol of L-Ala-COOMe was added and the mixture was stirred for 3 h, at the same conditions. After completion of the reaction, the magnetic nanoparticles were separated from the reaction mixture by an external magnet. The extraction process was performed by adding excess dry DCM to the mixture. Then, the DCM phase was evaporated by a rotary evaporator. The desired product was obtained as a powder and dried at room temperature.


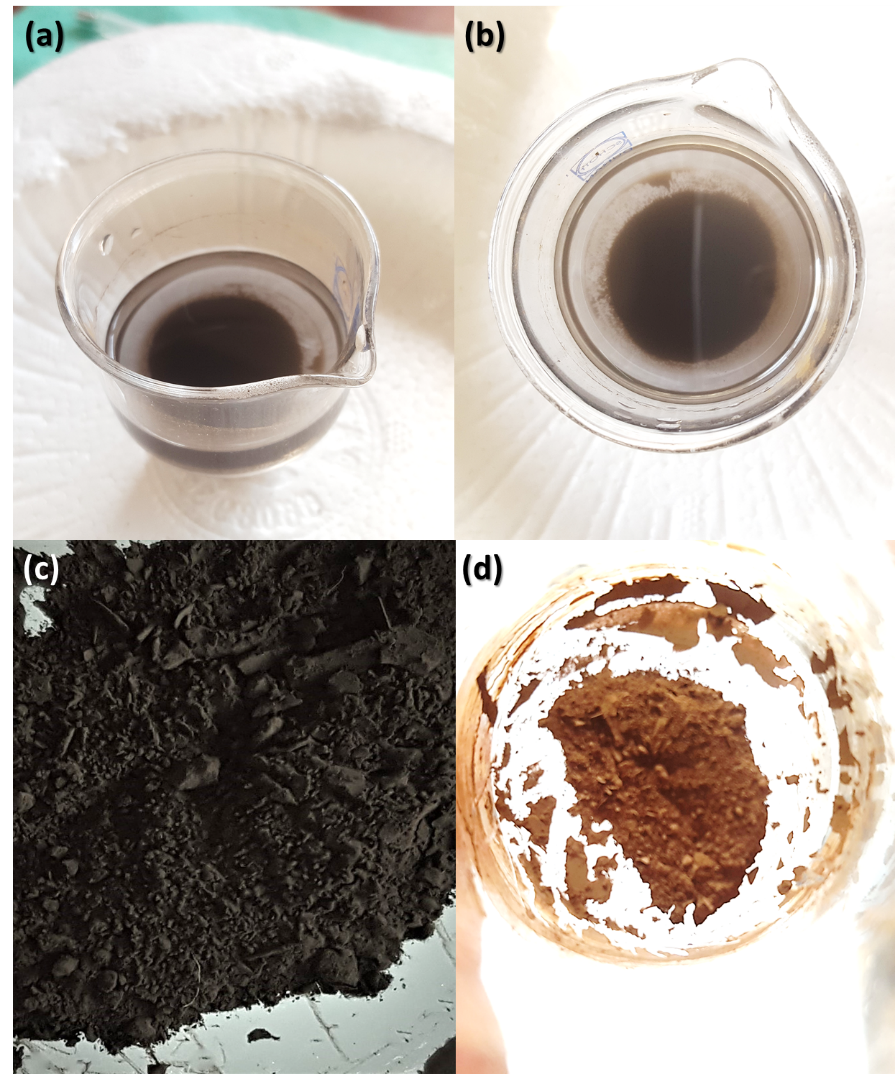


**Figure S9**. Digital images provided from fresh sample of Fe_3_O_4_@SiO_2_/TABHA particles in solution (a,b) and dried (c), and oxidized sample via exposure to the air (d).
